# The Effectiveness of Ultrasound Deep Heat Therapy for Adhesive Capsulitis: A Systematic Review and Meta-Analysis

**DOI:** 10.3390/ijerph19031859

**Published:** 2022-02-07

**Authors:** Jung-Ha Sung, Jung-Min Lee, Jung-Hyun Kim

**Affiliations:** 1 Department of Sports Medicine & Science, Graduate School of Physical Education, Kyung Hee University, Yongin-si 17104, Korea; jung.ha@khu.ac.kr (J.-H.S.); jungminlee@khu.ac.kr (J.-M.L.); 2 Department of Physical Education, Kyung Hee University, Yongin-si 17014, Korea; 3 Sports Science Research Center, Kyung Hee University, Yongin-si 17014, Korea; 4 Department of Sports Medicine, Kyung Hee University, Yongin-si 17014, Korea

**Keywords:** adhesive capsulitis, frozen shoulder, ultrasound therapy, deep heat therapy, systematic review, meta-analysis

## Abstract

Background: Adhesive capsulitis occurs with synovial inflammation and capsular fibrosis in the glenohumeral joint, leading to restriction of joint motion and pain. Heat therapy modalities, which aim at modifying dense collagenous tissues are commonly practiced interventions for patients with adhesive capsulitis; however, the effectiveness of ultrasound deep heat therapy (UST) on adhesive capsulitis is still unclear. Purpose: This systematic review and meta-analysis study was conducted to evaluate the effects of UST on the improvement of pain and glenohumeral joint function in adhesive capsulitis compared to (1) no treatment or placebo, and (2) any other therapeutic modalities. Methods: A literature search was carried out in January 2021 in Cochrane Central Register of Controlled Trials, PubMed, EMBASE, PEDro, Web of Science, and Google Scholar. The main outcomes of interest were self-reported pain scores, disability scores, and the range of motion. This study was conducted based on the guidelines of the PRISMA (Preferred Reporting Items for Systematic Review and Meta-Analyses) protocols, using random-effects models. Results: Seven studies were included in the systematic review with five studies forming the basis for meta-analyses. The effects of UST in patients with adhesive capsulitis were compared with placebo, shockwave therapy, corticosteroid injection, platelet-rich plasma injection, or cryotherapy. The results indicated that UST significantly improved pain scores when performed together with exercise and/or other physical modalities compared to placebo; however, whether UST provides benefits for the improvement of disability and/or the range of motion was uncertain in the present results. Conclusions: The present findings suggest that UST as a co-intervention combined with other physical modalities is an effective means of improving the overall pain in patients with adhesive capsulitis.

## 1. Introduction

Adhesive capsulitis of the shoulder (ACS), also called frozen shoulder or periarthritis of the shoulder, is caused by the abnormal process of synovial inflammation and capsular fibrosis in the glenohumeral joint. It leads to dysfunction of daily activities with pain and stiffness in the shoulder [1]. Tightness is present not only in the joint capsule, but also in other surrounding structures such as muscles, ligaments, and tendons. ACS occurs in approximately 3 to 5% of the general population, more frequently in women over 40 years old and in the prevalence of medical conditions such as diabetes mellitus, thyroid disease, or shoulder surgery. However, men tend to experience less improvement of clinical symptoms than women after treatment [2,3].

The primary ACS, also called idiopathic ACS, occurs without any specific trauma or history of injury. Meanwhile, secondary ACS occurs with an underlying etiology and/or associated pathologic conditions which are further categorized into intrinsic and extrinsic ACS. Intrinsic ACS is caused by a direct pathologic condition in the glenohumeral joint structures, such as fracture of the shoulder joint, calcific tendinitis, rotator cuff tendinopathy, or acromioclavicular arthritis. Extrinsic ACS is caused by conditions that are not directly related to the shoulder, such as cervical disc disease, thyroid disease, cerebrovascular disease, stroke, coronary artery disease, autoimmune disease, Dupuytren’s disease, or self-imposed immobilization. The symptoms of ACS can also occur as a complication after shoulder surgery [4,5].

ACS can be classified into four stages followed by the patient’s symptoms and duration [6]. In the first phase (i.e., in the early stage), patients complain of shoulder pain, especially at night in which inflammation occurs in the glenohumeral joint structures without significant adhesions or contractures. Early loss of external rotation motion which is an important sign in clinical examination of ACS may appear in this stage. In the second phase (i.e., after three months), synovial inflammation is pronounced with synovial proliferation resulting in early capsular adhesions and contracture. Patients experience worsening pain and gradually decreased shoulder range of motion in all directions. In the third phase (i.e., in 9 to 15 months), the formation of dense collagenous tissue within the capsule occurs, causing significant adhesions and stiffness in the glenohumeral joint. In the fourth phase (the chronic stage), a complete adhesion of the glenohumeral joint is observed and patients persistently experience stiffness with decreased pain in the shoulder. When pain is effectively controlled, some patients also show a slight improvement in mobility [1,2,4].

Patients with ACS often experience that their condition resolves on its own in approximately 1 to 3 years. However, previous studies indicated that approximately 20 to 50% of ACS cases eventually lead to chronic symptoms [2]. The main goal of treatment is to relieve pain and improve functional movement. There are various treatment options, but a standardized approach does not exist so the selection of treatment preferences among rehabilitative, pharmacological, and operative interventions is dependent on patients’ symptoms and disease progression [7]. Generally, patients are treated with non-operative management for the initial 4 to 6 months, such as physical therapy, manual technique, mobilization, corticosteroid injection, stretching, and/or exercises. If symptoms persist despite conservative treatment, operative management could be performed [8].

One of the commonly practiced muscle relaxation techniques for patients with ACS is thermo-therapy as heat increases tissue temperature and local blood flow, helping alleviate muscle and joint stiffness [9]. Thermo-therapy is classified into superficial or deep heat therapy. Superficial heat modality such as hot packs and moist heat increases the temperature of superficial tissues to a depth of approximately 1 cm while deep heat modality such as ultrasound, shortwave, and microwave diathermy target deeper tissues within 3–5 cm of the tissue surface [10,11,12]. Especially, ultrasound therapy (UST) commonly used for ACS treatment is known to create molecular vibration, which helps to break down dense collagenous tissues within the capsule [13]. UST can be performed at either 1 or 3 MHz for tissue heating [13]. The depth of penetration is usually based on ‘half-value depth’ which stands for the distance 50% of the therapeutic heat energy dissipated. Since UST is known to produce heat energy between 1 and 2 half-value depths, 1 MHz is used when targeting deeper tissues such as approximately 2.5 to 5 cm, whereas 3 MHz is often used for more superficial tissues to a depth from 0.8 to 1.6 cm. [13,14,15]. However, the effect of UST alone or in combination with other interventions on the treatment of ACS is not clear. The previous review found no additional effects of UST based on only one trial compared with placebo for ACS [16]. In contrast, a recent study suggested that performing UST effectively alleviates the painful symptoms in ACS patients with end-range mobilization [17].

Therefore, the purpose of this systemic review and meta-analysis was to systemically synthesize available evidence regarding the effectiveness of UST alone or in combination with other treatments, for the management of pain, mobility, and functional disability in patients with ACS.

## 2. Material and Methods

### 2.1. Literature Search

The present study was conducted based on the guidelines of the PRISMA (Preferred Reporting Items for Systematic Review and Meta-Analyses) protocols [18]. Clinical trials were first searched using the keywords (Table 1) in January 2021 from the Cochrane Central Register of Controlled Trials, PubMed, EMBASE, PEDro, Web of Science, and we also conducted a Google Scholar search for additional publications. All literature screened based on the review of literature titles and abstracts was imported to Zotero first, and then duplicates were removed.

### 2.2. Inclusion Criteria

The studies were selected by the following criteria and those that did not meet all the inclusion criteria were excluded from the present review: (1) The studies that enrolled patients with ACS for any duration; (2) The studies that compared thermal ultrasound therapy alone or in combination with any other intervention; (3) The goal of treatment was to improve pain, mobility, or functional disability for which outcome measures were attained in pain score, range of motion, and disability score, respectively.

### 2.3. Methodological Quality Assessment

Three reviewers independently assessed selected trials using a modified Downs and Black checklist [19]. The checklist consists of a total of 27 scoring items: reporting (items 1–10), external validity (items 11–13), internal validity bias (items 14–20), internal validity confounding (items 21–26), and power (items 27). Trials with a total score below 15 were excluded, and seven relevant studies were finally selected after the quality assessment [20]. Fleiss’ kappa value was also calculated to determine the inter-rater reliability of the assessment between the three reviewers [21].

### 2.4. Data Extraction

To synthesize each outcome data, we recorded the following characteristics: (1) participant characteristics including age, sex, and duration of symptoms; (2) intervention characteristics, including the type of treatment, duration of treatment, type of co-interventions, and (3) numerical outcome for the assessment of pain, mobility, or functional disability.

Outcome measurements included the following instruments:

- For the assessment of pain, pain scores measured with the visual analog scale (VAS) were included. We combined data of activity pain with unspecified pain for the analysis.
- For the assessment of mobility, the shoulder range of motion (Abduction, Flexion, Internal rotation, External rotation) measured with a goniometer was included [16].
- For the assessment of disability, the Shoulder Pain and Disability Index (SPADI) consisting of 5 pain questions and 8 disability questions were included [22].

Disability data from various self-reported instruments such as Quick DASH (Disabilities of the Arm, Shoulder, and Hand) and SDQ (Shoulder Disability Questionnaire) were qualitatively summarized due to differences in scoring methods between the instruments [23,24].

### 2.5. Data Synthesis

Due to heterogeneity in a type of co-intervention and treatment duration, the analysis was first classified by intervention types. The main outcome variables were the treatment effect on the changes in pain score, range of motion, and disability score. However, the changes in mean and standard deviation (SD) between baseline and endpoint were not reported in most trials. Therefore, the changes in group mean and SD were calculated based on the reported pre-/post-intervention values, using the following equations [25]:

$${\mathrm{Mean}}_{change}={\mathrm{Mean}}_{endpoint}-{\mathrm{Mean}}_{baseline}$$

$${\mathrm{SD}}_{change}=\sqrt{{\left({\mathrm{SD}}_{change}\right)}^{2}+{\left({\mathrm{SD}}_{endpoint}\right)}^{2}-2*r*{\mathrm{SD}}_{baseline}*{\mathrm{SD}}_{endpoint}}$$

where *r* represents the correlation coefficient and was set at 0.8 based on the Cochrane guideline for systematic reviews [26].

### 2.6. Statistical Analysis

To assess heterogeneity, we used I^2^ statistics which are classified as high (>75%), substantial (50–90%), moderate (30–60%), and low heterogeneity (0–40%) [15]. When the *p* < 0.1, the null hypothesis was rejected, and the random-effects model was used, which is also considered an appropriate test for varied study populations and intervention methods [27]. Outcome data of the studies were the continuous type using specific numerical measurements. In this case, mean differences and standardized mean differences (SMD; Cohen’s D) (i.e., effect size) were calculated [27]. The *p*-values were calculated with 95% confidence intervals. A standardized effect size was considered small (0.2–0.5), moderate (0.5–0.8), and large (>0.8) [28]. All analyses were performed using Revman 5.3 (Review Manager (RevMan) (Computer program), version 5.3. Copenhagen: The Nordic Cochrane Centre, The Cochrane Collaboration, London, UK, 2014).

## 3. Results

### 3.1. Literature Inclusion

**Figure 1:**
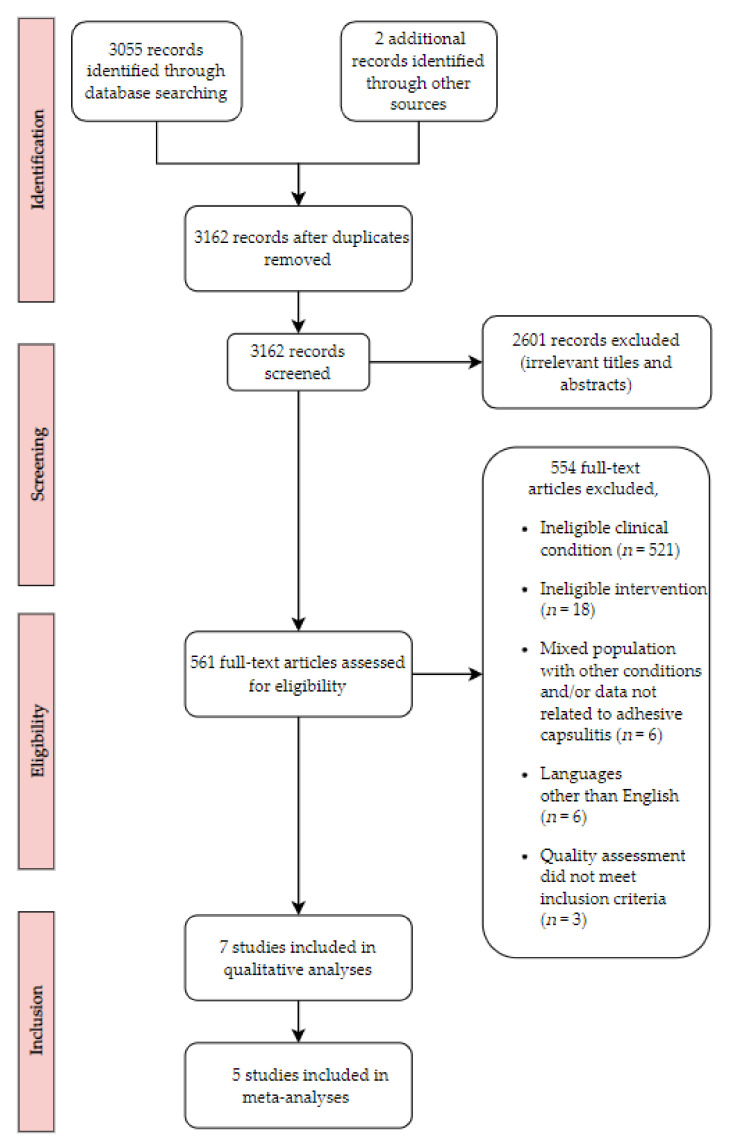
Flow chart for search strategy results.

After removing duplicates, 3055 records remained from which 2494 articles were further excluded due to the lack of relevancy upon the review of article titles. In addition, 561 articles were assessed through the review of article titles and abstracts. Furthermore, 551 articles were excluded since they did not meet eligibility criteria for participant characteristics, intervention types, or clinical disease conditions. Ten trials were selected with our inclusion criteria, but three trials did not meet our quality assessment criteria [29,30,31]. A total of seven trials were finally included for this systematic review and meta-analysis [23,24,32,33,34,35,36]. Specifically, seven trials were included for pain measurement using VAS. In the range of motion measurement, six trials for abduction and flexion were included. Five trials for internal rotation and external rotation were also included. Three trials were included for disability score. A flow diagram is presented in Figure 1.

### 3.2. Quality Assessment

Seven studies [23,24,29,30,31,32,33,34,35,36] were evaluated with a 27 question-modified Downs and Black checklist (Table 2). Fleiss’s kappa value was 0.93 for inter-rater reliability between the three reviewers. The ranges for this assessment were suggested by Landis and Koch: 0% or less (poor quality), 1–20% (slight quality), 21–40% (fair quality), 41–60% (moderate quality), 61–80% (substantial-quality), and 81% or greater (almost perfect quality) [37]. Three studies attempted to blind participants to the interventions they have received, and five studies reported that randomized participants to intervention groups (items 14 and 23). In addition, four studies tried to adjust the confounding factors such as difference in pre-treatment values of patients group or compliance of co-interventions (item 25). Most studies showed poor scores for the power section (item 27). Index of agreement in three raters for reliability using Fleiss’s kappa was classified as high quality.

### 3.3. Overview of the Studies

A total of 409 participants’ data from the seven trials published between January 2006 and November 2018 was included for the present analysis [23,24,32,33,34,35,36]. Fifty-three percent of participants were female except for one trial which was not reported [32]. The mean age of participants was approximately 52 years (27~77 years old) in this study except for one trial which was not reported. The range of the duration of symptoms was 1–9 months after excluding uncertain information in one study [36]. All studies reported the method of clinical assessment in patients. Patients in five studies were diagnosed by licensed physicians. In two studies, patients were diagnosed based on their clinical symptoms such as pain and passive ranges of motion [23,24]. For treatment modalities, three trials compared the UST with a sham condition. The two trials compared UST with shock wave therapy. The one trial compared UST with corticosteroid injection or platelet-rich plasma injection. Furthermore, the other trial compared UST with cryotherapy. All studies performed the main treatment with co-interventions such as exercises or mobilizations. The detailed information of co-intervention types is summarized in Table 3.

### 3.4. Meta-Analysis

For the meta-analyses, we focused on the improvement of the pain, range of motion, and disability score for which a total of five studies was included. In addition, we included data measured immediately after treatment duration to avoid the effects of confounding factors during the follow-up period.

For the following cases, we have qualitatively assessed the data because the outcomes of each comparison were suggested in only one trial. (1) UST versus corticosteroid injection, (2) UST versus platelet-rich plasma injection, (3) UST versus cryotherapy.

#### 3.4.1. Pain Score (VAS)

**Figure 2:**
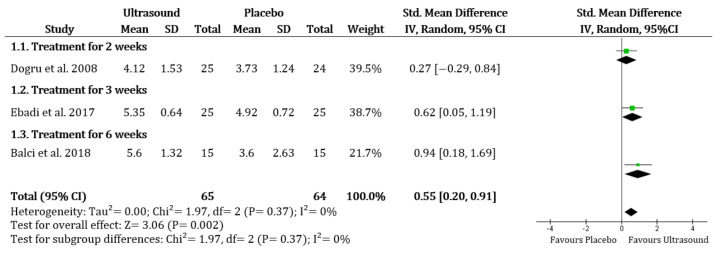
Overall pain: Ultrasound therapy versus placebo. Green square represents “Effect size”; Black diamond represents “Overall effect”.

Improvement in the pain score was significantly higher in the UST group than the placebo group (SMD 0.55, 95% CI 0.20 to 0.91; I^2^ = 0%) (Figure 2). In the comparison of UST versus shock wave therapy, there were no obvious differences between groups (SMD 0.27, 95% CI −0.91 to 1.45; I^2^ = 77%). There were no significant differences when comparing UST to corticosteroid injection or platelet-rich plasma injection (only based on Kothari et al. [23]). In addition, the improvement of pain was significantly higher in the UST group than in the cryotherapy group (only based on Ansari & Shah [33]).

#### 3.4.2. Range of Motion

**Figure 3:**
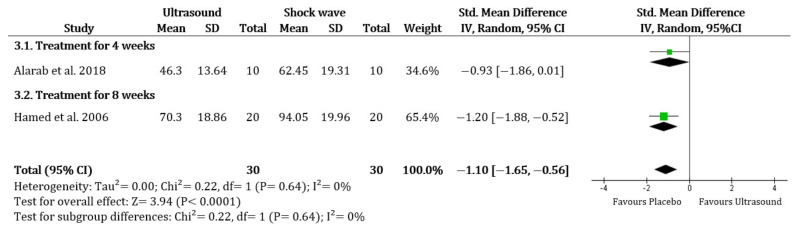
Range of motion (Flexion): Ultrasound therapy versus shock wave therapy. Green square represents “Effect size”; Black diamond represents “Overall effect”.

Comparing UST to placebo, there was no evidence that the UST was more effective for improvement of ABD (SMD 0.26, 95% CI −0.54 to 1.05; I^2^ = 79%), FL (SMD −0.14, 95% CI −1.11 to 0.84; I^2^ = 86%), IR (SMD −0.22, 95% CI −0.56 to 0.13; I^2^ = 0%) and ER (SMD −0.47, 95% CI −1.04 to 0.09; I^2^ = 59%). There was also no evidence that the UST was more effective than the shock wave therapy or the improvement of ABD (SMD –0.17, 95% CI −1.22 to 0.88; I^2^ = 72%), IR (only based on Alarab et al. [32]), and ER (only based on Hamed and El-Rahman [36]). However, there was a more significant change in shock wave therapy for FL (SMD −1.10, 95% CI −1.65 to −0.56; I^2^ = 0%) (Figure 3). There was no significant difference when comparing the UST group to the corticosteroid injection group or the platelet-rich plasma injection in all ROM (only based on Kothari et al. [23]).

#### 3.4.3. Disability Score

Immediately after the treatment, no significant differences were found when compared UST to placebo, corticosteroid injection, or platelet-rich plasma injection except for one trial which reported that there was more improvement of disability score in the placebo group than UST group (only based on Balci et al. [24]).

## 4. Discussion

The purpose of this systematic review and meta-analysis was to evaluate the effectiveness of the UST modality on the improvement of clinical outcomes in patients with ACS. To the best of our knowledge, this study is the first review to specifically examine the benefits of UST with other interventions for ACS. The ACS patients in all studies included in this review showed significant improvement in their symptoms after performing UST when combined with exercise, mobilization, or other physical modalities. However, whether there are additional benefits of the UST when carried out with other therapeutic interventions is still ambiguous.

The main finding of the study is that UST compared to placebo is an effective intervention when carried out with exercise and/or other physical modalities. Dogru et al. [34] reported that the UST group showed greater improvement in FL, IR, and ER than the sham UST group without any difference in pain improvement between groups. However, one drawback of the study was that both groups had different baseline mobility (i.e., ROM) scores, and the compliance rate for the home exercise programs was also different between the groups, which may have greatly influenced the results. Similarly, Ebadi et al. [35] and Balci et al. [24] found no evidence that UST has additional effects along with exercise or other physical modalities. Interestingly, after normalizing the point of measurements and treatment duration, the present study found significant pain improvements in the UST group compared to the sham UST group. This finding is similar to the previous study that UST effectively reduced pain compared to cryotherapy in patients with ACS [33].

Some mechanisms have been proposed that might explain the therapeutic effects of UST for ACS patients, which include thermal and non-thermal effects. Non-thermal effects are generally aimed at acute injury or tissue healing. One of the non-thermal treatment effects is known to be molecular vibration which forms cavitation and microstreaming by inducing cell membrane permeability and soft tissue healing. Interestingly, molecular vibration (i.e., mechanical effects) turns into heat generation, which increases the extensibility of collagen and tendon. Therefore, UST is expected to alleviate the viscosity of the collagen and resolve fibrosis, leading to pain reduction and mobility improvement in ACS patients [14,34,35]. Furthermore, UST elevates the threshold for free nerve activation by delivering heat in large diameter myelinated nerve fibers, which is thought to alleviate the pain based on the gating mechanism [36,38].

It should be noted that a comprehensive assessment of changes in disability scores was not possible due to the utilization of various instruments for evaluating disability in previous studies. Evaluating results only measured immediately after the treatment duration, the two studies reported conflicting evidence regarding the effects of UST on improving disability scores compared to placebo. These conflicting results may have resulted from different dosages and sessions of treatment between the two trials. For example, Balci et al. [24] treated ACS patients at a frequency of 1 MHz while Dogru et al. [34] treated at 3 MHz, which is known to transfer heat through the tissue faster than 1 MHz. The 3 MHz mode, which is typically applied to the 2.5 cm or less, generates energy absorbed in the superficial tendon with little effect on the bone, whereas the 1 MHz mode used for 2.5–5 cm deep delivers much greater energy to the bone. Therefore, the dosage must be considered according to treatment goals [14]. Furthermore, Balci et al. [24] treated patients with 18 sessions for six weeks, whereas Dogru et al. [34] performed UST more frequently such as 10 sessions for two weeks. Future studies with similar therapeutic regimens are warranted to compare the additional benefits of UST for the improvement of disability in ACS patients.

The present study provides new insights about the effectiveness of ultrasound deep heat therapy for adhesive capsulitis, but it does have some limitations. The main limitation of the present study is the limited number of data syntheses due to the various types of interventions applied for the comparison between UST and other modalities. For example, two trials included in our review that compared UST with shock wave therapy have reported conflicting results. Alarab et al. [32] reported that UST was less effective in pain relief and ROM improvement compared to extracorporeal shock wave therapy. In contrast, Hamed and El-Rahman [36] reported that UST was an effective means of relieving pain compared to piezoelectric shock wave therapy. These conflicting results may have been caused by different mechanisms of the shock wave therapy for heat energy generation between the trials. Additionally, due to a paucity of previous results, it is unclear whether UST is more effective than corticosteroid injection, platelet-rich plasma injection, or cryotherapy. One trial found no additional effects of UST compared to corticosteroid injection or platelet-rich plasma injection except for the improvement in ER [23]. Meanwhile, Ansari and Shah reported that UST therapy has a greater benefit for pain relief than cryotherapy [33].

Despite the above-mentioned limitations, the main strength of the present study is that the additive effect of the UST with other physical therapy, exercises, and/or mobilization compared with placebo or other treatment approaches for ACS was analyzed through the recently available data. Currently, there is no established standard of care for the management of ACS, and the clinical utility of the UST is still ambiguous. All studies included in this review performed UST therapy together with at least one exercise program. The reason is thought to be that the increase in tissue temperature and the viscoelasticity caused by ultrasound therapy is further strengthened by performing an exercise. Noten et al. [39] found that the effect of high-grade mobilization was superior to low-grade mobilization in patients with primary ACS. Griggs et al. [40] showed evidence that stretching exercise programs improve the symptoms in patients with second phase ACS. Recent research also suggested that rotator cuff strengthening exercises could reinforce the stability and functional ability of the shoulder to maintain normal dynamic movement with sufficient force [41]. However, the tissue irritability at different phases of ACS should be considered for prescribing exercise programs, as excessive shoulder movement may exacerbate symptoms, especially during the inflammatory phase [5,33]. Therefore, based on the present evidence, we recommend the inclusion of UST accompanied by exercise programs such as gentle stretching, mobilization, and shoulder strength exercise for the effective management of pain in ACS patients.

## 5. Conclusions

The present study adds to the growing body of literature by utilizing recent trials to evaluate the effects of deep heat UST for ACS. We found that the UST accompanied by co-interventions is an effective means for the improvement of the overall pain. However, when compared to other treatment modalities together with exercise or physiotherapy, no additional benefits were found except for cryotherapy. In the future, high-quality and well-designed studies comparing the sole and additive effects of UST at various stages of ACS compared with other non-operative interventions are needed to draw definitive conclusions and assist treatment practice.

## Figures and Tables

**Table 1:** Search keywords.

Keywords	
Population	(adhesive capsulitis OR frozen shoulder OR periarthiritis shoulder OR shoulder stiffness)
AND Intervention	((ultrasound OR ultrasonic) AND (therapy OR therapeutic) NOT (guided OR imaging))
AND Outcome	(pain OR range of motion OR disability)

**Table 2:** Quality assessment scores. (Modified Downs and Black checklist).

Assessment Items	Study						
	Hamed et al., 2006	Dogru et al., 2008	Ansari et al., 2013	Ebadi et al., 2017	Kothari et al., 2017	Alarab et al., 2018	Balci et al., 2018
Reporting (11)	9	10	9	11	11	8	10
External validity (3)	2	3	3	3	3	2	1
Internal validity bias (7)	4	6	4	6	5	5	6
Internal validity confounding (6)	3	4	3	4	4	4	4
Power (1)	0	1	0	0	0	0	0
Total score (28)	18	24	19	24	23	19	21

Total score: excellent (26–28); good (20–25); fair (15–19); and poor (≤14).

**Table 3:** Main characteristics of populations, interventions, and outcome measures of included randomized trials.

Study	Sample0020 (Age, Group, Exercise Type)	Gender (M/F)	Duration of Symptoms	Treatment Duration (Follow-Up) and Dosage	Outcome Measures	Main Finding
Hamed et al., 2006	Age= 50 ± 3.4 years PSWT (*n* = 20), UST (*n* = 20) Mobilizing exercise	PSWT: 8/12 UST: 4/16	>3 month	24 sessions for 8 week 3 MHz	Pain (VAS) ROM (ABD, FL, ER)	↓Pain ↑ROM
Dogru et al., 2008	Age= 55.3 ± 7.6 years UST (*n* = 25), Sham UST (*n* = 24), Mobilizing exercise with superficial heating	UST: 11/14 SHAM: 10/14	>5 month	10 sessions for 2 week (3 month) 3 MHz, 1.5 W/cm^2^	Pain (VAS, SPADI) ROM (ABD, FL, IR, ER); Disability (SPADI, QoL)	⇔ Pain ⇔ Disability; ↑ROM (except ABD)
Ansari et al., 2013	Age= 53.8 ± 3.9 years UST (*n* = 20), CTP (*n* = 20) End range mobilization exercise	UST: 5/15 CTP: 5/15	>2 month	24 sessions for 4 week Pulsed 1.5 W/cm^2^	Pain (VAS)	↓Pain
Ebadi et al., 2017	Age= 49.7 years UST (*n* = 25), Sham UST (*n* = 25) Stretching & strength exercise	UST: 10/15 SHAM: 10/15	>5 month	10 sessions for 3 week (3 month) 3 MHz, 1.5 W/cm^2^	Pain (VAS) ROM (ER, IR, ABD, FL) Functional disability (Oxford Shoulder Score)	⇔ Pain ⇔ ROM ⇔ Disability
Kothari et al., 2017	Age= 27–75 years PRP (*n* = 62), CS (*n* = 60), UST (*n* = 58) Home exercise	PRP: 34/28 CS: 29/31 UST: 23/35	>1 month	7 sessions for 2 week (3, 6, 12 week) 1 MHz, 1.5 W/cm^2^	Pain (VAS) ROM (ABD, FL, ER, IR, EX)Functional disability (DASH)	↓Pain ↑ROM ↓Disability
Alarab et al., 2018	Age= 45.3 ± 8.6 years ESWT (*n* = 10), UST (*n* = 10) Mobilizing exercise	Unknown	2–9 month	12 sessions for 4 week 3 MHz, 1.0 W/cm^2^	Pain (VAS) ROM (ABD, FL, IR)	↓Pain ↑ROM
Balci et al., 2018	Age= 55.7 ± 8.2 years UST (*n* = 15), Sham UST (*n* = 15) Pendulum, stretching, isometric, resistant exercises with superficial heating	UST: 7/8 SHAM: 7/8	>3 month	6 week (24 week); 18 sessions 1 MHz, 1.5 W/cm^2^	Pain (VAS) ROM (ABD, FL, IR, ER) Functional disability (UCLA, SDQ)	⇔ Pain ⇔ ROM ⇔ Disability

UST, ultrasound; PSWT, piezoelectric shock wave therapy; CTP, cryotherapy; PRP, platelet-rich plasma; CS, corticosteroid injection; ESWT, extracorporeal shock wave therapy; PSWT, piezoelectric shock wave therapy; ABD, abduction; FL, flexion; IR, internal rotation; ER, external rotation; EX, extension; VAS, visual analog scale; SPADI, shoulder pain, and disability index; QoL, quality of life; UCLA, University of California and Los Angeles shoulder scale; SDQ, shoulder disability questionnaire; DASH, disabilities of the arm, shoulder, and hand score. Downward arrow represents “Increase”; Upward arrow represents “Decrease”; Bidirectional arrow represents “No change”.

## Data Availability

No new data were created or analyzed in this study. Data sharing is not applicable to this article.
